# Occurrence of clinically relevant thyroid nodules in adults

**DOI:** 10.2478/raon-2026-0001

**Published:** 2026-01-13

**Authors:** Katja Zaletel, Katja Tuta, Tina Usaj, Katica Bajuk Studen, Natasa Bedernjak Bajuk, Miha Jesenko, Tanja Radevska, Edvard Pirnat, Matej Gregoric, Urska Blaznik, Masa Hribar, Igor Pravst, Simona Gaberscek

**Affiliations:** Division of Nuclear Medicine, University Medical Centre Ljubljana, Ljubljana, Slovenia; Faculty of Medicine, University of Ljubljana, Ljubljana, Slovenia; National Institute of Public Health, Ljubljana, Slovenia; Nutrition Institute, Ljubljana, Slovenia; Biotechnical Faculty, University of Ljubljana, Ljubljana, Slovenia

**Keywords:** thyroid nodule, nationally representative population, women of reproductive age, pregnant women

## Abstract

**Background:**

We aimed to comprehensively investigate the occurrence of thyroid nodules in a nationally representative population as well as in women of reproductive age from a geographic area with adequate iodine intake over the last two decades.

**Patients and methods:**

This prospective cross-sectional study included 653 adult participants from three groups: a nationally representative gender-mixed group (205 participants) and women of reproductive age, including non-pregnant (306 participants) and pregnant (142 participants) women. For each participant, demographic data were collected, thyroid-stimulating hormone (TSH) levels were measured, thyroid volume was estimated, and the presence and size of thyroid nodules were recorded with high-resolution ultrasound. The ultrasound characteristics were analysed.

**Results:**

Among the nationally representative participants, nodules were detected in 44.9%, with 39.0% larger than 5 mm and 13.7% larger than 0.5 mL. Among women of reproductive age, nodules were detected in 22.5%, with 14.1% larger than 5 mm and only 2.0% greater than 0.5 mL. The prevalence and size of nodules increased significantly with age in all groups, being significantly lower in non-pregnant women than in pregnant women, who were also older. In non-pregnant women of reproductive age, the number of nodules increased significantly after the age of 25, with the number of nodules larger than 5 mm increasing only after the age of 40.

**Conclusions:**

Thyroid nodules are prevalent in the population, but are rarely clinically significant. Therefore, screening for thyroid nodules in asymptomatic individuals with normal thyroid findings on clinical examination should be avoided.

## Introduction

In recent decades, the frequency of ultrasound (US) examination of thyroid morphology has notably increased the detection of thyroid nodules, many of which are clinically insignificant and do not require treatment.^1^ The majority of asymptomatic nodules with unsuspicious US characteristics or benign cytology show no growth even within 5 years.^2,3^ However, imaging procedures performed for unrelated conditions, most notably neck US and vascular Doppler, and to a lesser extent computed tomography, magnetic resonance imaging and positron emission tomography with fluorode-oxyglucose, remain important sources of incidental asymptomatic thyroid findings.^4^ The estimated prevalence of thyroid nodules on US imaging varies considerably, ranging from 20% to 68%. This variation depends primarily on the demographic characteristics of the group studied, the sensitivity of US equipment for detecting thyroid nodules, and iodine supply in the population studied.^5,6^

With age, both the prevalence of thyroid nodules in the population and the size of nodules increase significantly, although the likelihood of clinically relevant nodules being malignant tends to decrease.^6–8^ Thyroid nodules have been reported to correlate with body mass index (BMI) and to be more prevalent in women.^8,9^ During pregnancy, they have been observed in up to 30% of women^10^, with both the number and size of nodules increasing notably by the third trimester.^11^ In addition, the detection of thyroid nodules is significantly influenced by the characteristics of the US equipment. Transducers with higher frequency and better resolution often lead to a significant increase in the number of detected nodules.^5^

When it comes to iodine supply, iodine deficiency has been associated with a higher prevalence of nodules.^12^ Furthermore, even in the range of adequate iodine intake, the prevalence tends to be higher when iodine intake was lower.^13^ After iodine fortification, a reduction in thyroid volume and a tendency to reduce the number of thyroid nodules was observed.^14^ In Slovenia, which was previously mildly iodine deficient, a 2.5-fold increase in mandatory iodization of salt in 1999 led to a significant improvement in mean urinary iodine concentrations, which reached 148 μg/L in school-aged children and 176 mg/g creatinine in pregnant women, both indicating adequate iodine supply.^15,16^ Although there was a significant decrease in the incidence of diffuse goiter in the first decade after iodine supplementation was increased, we found a more than 1.5-fold increase in the incidence of thyroid nodules; this increase was primarily attributed to the increasing frequency of US examinations of the neck.^15^

Although thyroid nodules are commonly encountered in clinical practice, research on the prevalence of this pathology has been relatively limited over the past decade, when the use of high-resolution US imaging has become widespread. Therefore, we aimed to comprehensively investigate the prevalence and clinical significance of thyroid nodules in both a nationally representative population and in women of reproductive age, including pregnant women, residing in an area with adequate iodine intake over the past two decades.

## Patients and methods

### Study population

**FIGURE 1.**
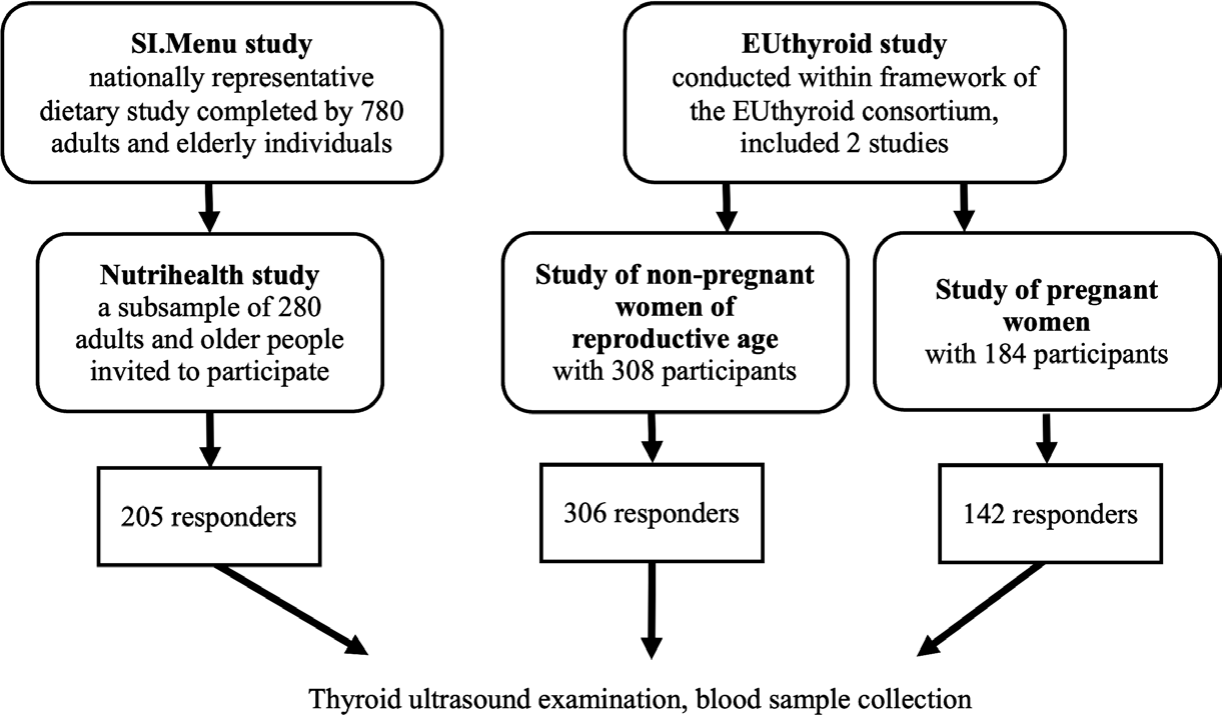
Study population flowchart.

The study population was drawn from two single-centre, prospective, cross-sectional surveys conducted between 2017 and 2018: the Nutrihealth study and the EUthyroid study (Figure 1).

The Nutrihealth study was an extension of the national cross-sectional food consumption study SI.Menu, which was conducted on a nationally representative sample of adolescents, adults and older people, as previously published.^17^ The SI.Menu recruitment period was from March 2017 to February 2018, and of the 780 adult and older participants who completed the study, a subsample of 280 adult and older subjects participated in the Nutrihealth study. The recruitment period for this sub-sample was from June 2017 to September 2018. Altogether, 205 of 280 participants responded to invitation for participation in the US examination of the thyroid gland. All participating subjects gave their informed consent. The study was approved by the Slovenian National Medical Ethics Committee (identification number 0120-337/2016-4) and registered at ClinicalTrials.gov (NCT03284840).

The EUthyroid study was conducted as part of the EUthyroid consortium, whose main objective was to collect data on iodine status from 48 European studies.^18^ Between October 2017 and July 2018, two groups of healthy female volunteers aged 18 to 49 years without known thyroid disease were recruited from different regions of Slovenia. We included 308 non-pregnant women of reproductive age and 184 pregnant women. However, the group of participants was not nationally representative. In the final phase, 306 of 308 women of reproductive age and 142 of 184 pregnant women participated in the US examination of the thyroid gland. All women gave informed consent and the study was approved by the Slovenian National Medical Ethics Committee (identification number 0120-508/2017-2).

### Study protocol

We examined a total of 653 participants at the Outpatient Department for Thyroid Diseases at the Division of Nuclear Medicine, University Medical Centre Ljubljana. In the Nutrihealth group, demographic data were collected, including a known history of thyroid disease and information on thyroid medication, and BMI was calculated (body weight (kg)/height^2^(m^2^)). In pregnant women, data on week of gestation and BMI were recorded. Thyroid morphology was assessed by high-resolution US examination and all participants also provided a blood sample. All participants with a nodule volume greater than 0.5 mL were invited for additional diagnostic examinations to further evaluate the malignancy risk of the nodule. In selected cases additional procedures included technetium-99m pertechnetate scintigraphy and US-guided fine-needle aspiration biopsy with cytological examination of the sample.

### Thyroid ultrasound

The US examination of the thyroid gland was performed using a Samsung HS70A device (Samsung Medison) equipped with a 4-12 MHz linear multifrequency transducer to achieve optimal resolution of both superficial and deeper thyroid structures. Thyroid volume and the presence and number of thyroid nodules were assessed. First, we identified all nodules, defined as any spherical or ellipsoidal lesion in the thyroid gland. Following the European Thyroid Association (ETA) guidelines for US malignancy risk stratification of thyroid nodules in adults, we then assessed the presence of nodules at least 5 mm in any dimension.^19^ In addition, we assessed the presence of a dominant nodule with a volume greater than 0.5 mL, corresponding to a nodule size of at least 10 mm in all three dimensions.

The volume of each thyroid lobe and the volume of the dominant thyroid nodule were calculated using the ellipsoid model (width x length x thickness x (/6)). All measurements were performed by one of two experienced investigators, each of whom had more than 15 years of experience in thyroid US. As previously reported, the inter-observer coefficient of variation was approximately 10%, while the intra-observer coefficient of variation was less than 10%.^16^

### Laboratory tests

The concentration of thyroid-stimulating hormone (TSH) was measured using a commercially available kit (the TSH3-Ultra assay) on the Advia Centaur XP Immunoassay System (Siemens Healthineers). The detection limit of the assay is 0.008 mIU/L, with a linearity range from 0.008 to 150.0 mIU/L and a recovery range between 96.3% and 105.0%. The normal reference range for TSH was 0.59 to 4.23 mIU/L.^20^

### Statistical analysis

The normality of continuous variables distribution was assessed using the Kolmogorov-Smirnov test. As multiple variables were not found to follow a normal distribution, data are presented as median (range) for most variables. The number of nodules per person is presented as mean with associated range. Groups comparisons were performed using the nonparametric Mann-Whitney test, and correlations were assessed using Spearman’s rho correlation coefficient. Categorical variables were compared using Chi-squared test. Statistical analyses were conducted with MedCalc Statistical Soft-ware version 22.026 (2024 MedCalc Software). Statistical significance was set at a threshold of p < 0.05.

## Results

### Characteristics of study population

The characteristics of the Nutrihealth participants are shown in Table 1. Thyroid glands were significantly smaller in women than in men (p < 0.001). Thyroid nodules were present in 44.9% of participants, which was not significantly higher than the 39% prevalence of nodules larger than 5 mm in any dimension. In contrast, the proportion of nodules greater than 0.5 mL was significantly lower at 13.7% (p < 0.001 for both comparisons). Although the proportion of subjects with nodules did not differ significantly between genders, the mean number of nodules per subject with nodules was significantly higher in women (p = 0.017). Otherwise, the two genders did not differ significantly in terms of age, BMI, dominant nodule volume or TSH concentration. In the Nutrihealth group, 7/205 (3.4%) participants reported a history of thyroid disease and 4/205 (2.0%) were taking thyroid medication.

**TABLE 1.** Clinical characteristics of nationally representativo Nutrihealth study group

	All N = 205	Women N = 126 (61.5%)	Men N = 79 (38.5%)	p-value
Age, years (median, range)	62 (18–74)	60 (20–74)	64 (18–74)	0.177
BMI, kg/m^2^ (median, range)	26.3 (17.9–43.4)	25.5 (17.9–43.4)	26.8 (18.2–41.7)	0.088
Thyroid volume, mL (median, range)	8.15 (2.0–46.7)	6.8 (2.0–46.7)	10.8 (2.0–28.4)	<0.001
Proportion of subjects with nodules (number, %)	92 (44.9)	60 (47.6)	32 (40.5)	0.069
Proportion of subjects with nodules > 5 mm (number, %)	80 (39.0)	52 (41.3)	28 (35.4)	0.058
Proportion of subjects with nodules > 0.5 mL (number, %)	26* (13.7)	18* (14.3)	8* (10.1)	0.385
Number of nodules per person with nodules (mean, range)	2.3 (1–6)	2.5 (1–6)	1.8 (1–6)	0.017
Dominant nodule volume, mL (median, range)	0.28 (0.02–19.00)	0.30 (0.02–19.00)	0.19 (0.02–17.20)	0.785
TSH, mIU/L (median, range)	8.15 (2.0–46.7)	2.04 (0.13–9.52)	1.88 (0.26–5.39)	0.056

1 BMI = body mass index; N = number; p-value = comparison between women and man (Mann-Whitney test for continuous variables and Chi-squared test for categorical variables TSH = thyroid-stimulating hormone (normal range, 0.59–4.23 mIU/L)

* = p < 0.001 compared to proportion of all subjects with nodules and proportion of subjects with nodules> 5 mm in each study group (Chi-squared test)

In the EUthyroid study group (Table 2), women of reproductive age were significantly younger (p < 0.001), and their thyroid volume was not significantly different from that of pregnant women. Thyroid nodules were present in 22.5% of participants, with a significantly higher proportion in pregnant women (p = 0.031). Nodules larger than 5 mm were detected in 14.1% of EUthyroid participants, which was significantly lower than the overall proportion of nodules (p < 0.001), but there was no significant difference between subgroups. Nodules greater than 0.5 mL were found in only 2.0% of participants, which was significantly lower than the proportion of all nodules and those larger than 5 mm (p < 0.001 for both comparisons). They were also significantly more common in pregnant women (p = 0.023), who also had a significantly higher number of nodules per person (p = 0.036). The volume of the dominant nodule did not differ significantly between the subgroups. However, the TSH concentration was significantly higher in women of reproductive age than in pregnant women (p < 0.001).

**TABLE 2.** Clinical characteristics of EUthyroid study group

	All N = 448	Women of reproductive age N = 305 (68.0%)	Pregnant women N = 142 (32.0%)	p-value
Age, years (median, range)	30 (19–56)	27 (19–49)	33 (20–46)	< 0.001
Thyroid volume, mL (median, range)	7.4 (2.5–30.5)	7.3 (2.5–16.6)	7.4 (3.3–30.5)	0.417
Proportion of all women with nodules (number, %)	101 (22.5)	60 (19.7)	41 (28.9)	0.031
Proportion of women with nodules > 5 mm (number, %)	63** (14.1)	37** (12.1)	26** (18.3)	0.082
Proportion of women with nodules > 0.5 mL (number, %)	9* (2.0)	3* (1.0)	6* (4.2)	0.023
Number of nodules per person with nodules (mean, range)	1.5 (1–6)	1.3 (1–4)	1.8 (1–6)	0.036
Dominant nodule volume, mL (median, range)	0.09 (0.02–3.83)	0.08 (0.02–2.46)	0.12 (0.03–3.83)	0.084
TSH, mU/L (median, range)	1.55 (0.27–116.90)	1.61 (0.32–116.90)	1.46 (1.48–4.38)	< 0.001

1 N = number; p-value = comparison between women of reproductive age and pregnant women (Mann-Whitney test for continuous variables and Chi-squared test for categorical variables); TSH = thyroid-stimulating hormone (normal range, 0.59−4.23 mIU/L)

* = p < 0.001 compared to proportion of all women with nodules and proportion of women with nodules > 5 mm in each study group (Chi-squared test)

### Thyroid volume

The thyroid volume of both EUthyroid women of reproductive age and pregnant women did not differ significantly from that of the women in the Nutrihealth group. However, it was significantly smaller compared to the overall Nutrihealth group (p < 0.001 and p = 0.024, respectively) and the subgroup of Nutrihealth men (p < 0.001 for both comparisons).

As shown in Table 3, thyroid volume was significantly correlated with both the number of nodules and the volume of the dominant nodule in the Nutrihealth participants. In the male participants, there was also a correlation with BMI. In all Nutrihealth participants, thyroid volume was negatively correlated with TSH levels, but no correlation with age was observed. However, in EUthyroid women of reproductive age, thyroid volume correlated with age. In both Euthyroid subgroups, thyroid volume correlated with the number of nodules, but not with the volume of the dominant nodule. It also showed a negative correlation with TSH concentration. In pregnant women, a correlation with BMI was also observed, but there was no correlation with trimester or week of gestation.

**TABLE 3.** Correlation of thyroid volume with age, body mass index (BMI), nodule characteristics, and thyroid-stimulating hormone (TSH)

	Nutrihealth women N = 126		Nutrihealth man N = 79		EUthyroid women of reproductive age N = 305		EUthyroid pregnant women N = 142	
	rho	p-value	rho	p-value	rho	p-value	rho	p-value
Age	0.100	0.266	-0.065	0.569	0.134	0.019	0.101	0.231
BMI	-0.011	0.911	0.242	0.039	n.a.	n.a.	0.404	0.001
Number of nodules	0.416	< 0.001	0.337	0.002	0.116	0.042	0.185	0.028
Number of nodules > 5 mm	0.416	< 0.001	0.320	0.004	0.156	0.006	0.290	< 0.001
Volume of dominant nodule	0.390	0.004	0.553	0.002	0.059	0.727	0.251	0.217
TSH	-0.269	0.005	-0.509	< 0.001	-0.151	0.008	-0.255	0.002

1 N = number; n.a., not applicable; rho = Spearman’s rank correlation coefficient

### Thyroid nodules

**FIGURE 2.**
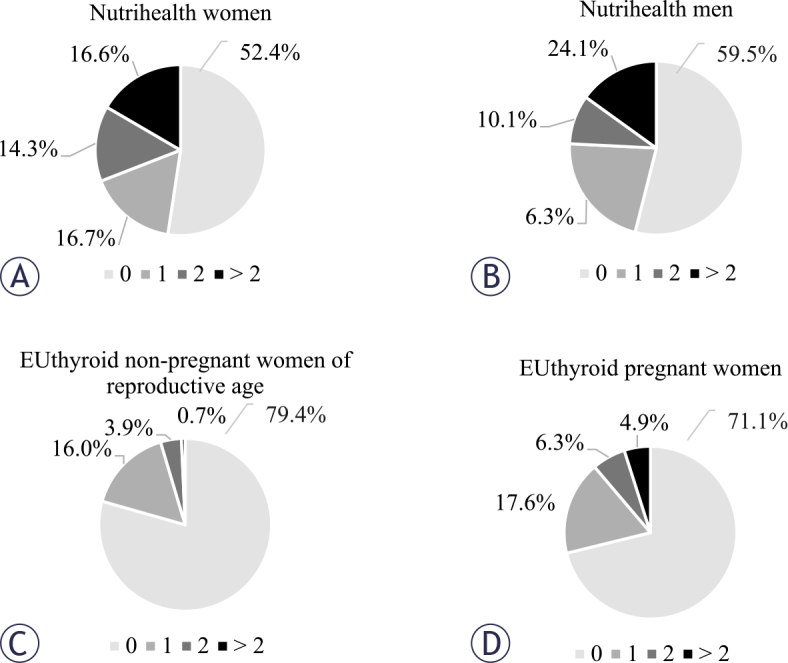
Distribution of participants with respect to the number of thyroid nodules.

We identified a total of 207 nodules in the Nutrihealth study, of which 150 were found in women and 57 in men. Among the participants, 113/205 (55.1%) had no nodules, 40/205 (19.5%) had 1 nodule, 26/205 (12.7%) had 2 nodules, and 26/205 (12.7%) had 3 or more nodules. Figure 2 (a. and b.) shows the distribution of female and male participants based on the number of thyroid nodules. The number of nodules increased significantly with age in both women (rho = 0.411, p < 0.001) and man (rho = 0.329, p = 0.003). Those without nodules were significantly younger (median, 55 years *vs*. 66 years, p < 0.001) and had a significantly higher TSH concentration (median, 2.04 mIU/L *vs*. 1.94 mIU/L, p = 0.045). BMI did not differ significantly between those with and without nodules, although a significant correlation of BMI with the number of nodules larger than 5 mm was confirmed (rho = 0.165, p = 0.029).

In the EUthyroid study, we identified a total of 154 nodules, 81 of which were in women of reproductive age and 73 in pregnant women. In this group, the proportion of participants with nodules was significantly lower than in the Nutrihealth group (22.5% and 44.9% respectively, p < 0.001). The distribution of participants in terms of the number of thyroid nodules is shown in Figure 2 (c. and d.). The number of nodules increased significantly with age in both women of reproductive age (rho = 0.117, p = 0.041) and pregnant women (rho = 0.201, p = 0.016). EUthyroid non-pregnant women of reproductive age without nodules were significantly younger (median, 27 years and 30 years, respectively, p = 0.006) and had higher TSH concentrations (median, 1.64 mIU/L and 1.35 mIU/L, respectively, p = 0.016). In pregnant women, no significant differences in age, BMI or TSH levels were observed in relation to the presence of thyroid nodules.

**FIGURE 3.**
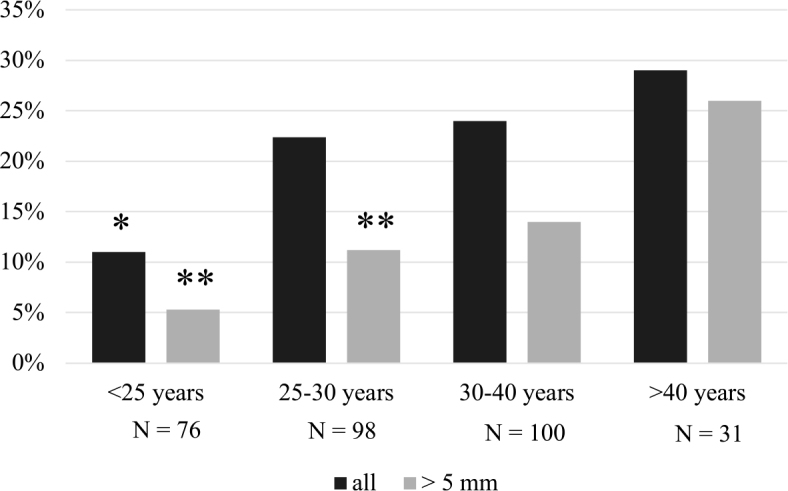
The proportion of all nodules and nodules > 5 mm in EUthyroid nonpregnant women of reproductive age. * = p < 0.05 compared to all older age groups (Chi-squared test) ** = p < 0.05 compared to the age group > 40 years (Chi-squared test)

In EUthyroid non-pregnant women of reproductive age, we also examined the number of nodules at different age periods. We found that the total number of nodules was significantly lower in individuals younger than 25 years compared to older individuals. In addition, the number of nodules larger than 5 mm increased significantly after the age of 40 (Figure 3).

### Clinical outcome in participants with nodules > 0.5 mL

Altogether, 35 participants with a dominant nodule volume greater than 0.5 mL were invited for further evaluation of malignancy risk, including 26 from the Nutrihealth study and 9 from the EUthyroid study. Among the 27 participants who responded, thyroid autonomy was confirmed in 4 patients, cytology reports were unsuspicious in 20 patients, and additional diagnostic procedures were not performed in 3 patients due to an unsuspicious US appearance. No malignant lesions were identified.

## Discussion

Our study shows that despite a high prevalence of nodules in the population, the proportion of those exceeding 5 mm in at least one dimension is significantly lower in younger women of reproductive age. Given our finding that the proportion of clinically significant nodules of 0.5 mL or more is only 13.7% in the general population and 2% in women of reproductive age, and given the previously reported prevalence of thyroid cancer of less than 10%,^19^ thyroid nodule screening should be discouraged, as also recommended in the most recent ETA guidelines.^1^

Earlier estimates of the prevalence of thyroid nodules were often based on retrospective assessments of selected, nonrepresentative populations performed by different investigators using low-resolution US equipment. In one of the earliest studies of participants aged 19–50 years living in an area not known for endemic goiter, thyroid nodules were confirmed in 21% of individuals.^5^ Later, nodules larger than 5 mm were found in 22% of participants aged 18–65 years living in an iodine-deficient area.^21^ In a later study conducted in the same area, involving participants aged 19–93 years and using high-resolution US equipment, nodules larger than 5 mm were found in 31% of cases. However, the overall prevalence of nodules was 68%, with 42% occurring in those under 40 years of age and 76% in those over 60 years of age.^6^ A recent large retrospective evaluation of subjects undergoing high-resolution thyroid US as part of screening examinations found a prevalence of thyroid nodules of 34%, ranging from 13% in those under 30 years of age to 55% in those over 70 years of age.^8^ While our data in a nationally representative population also confirmed a high prevalence of thyroid nodules, including those larger than 5 mm, our finding of a significantly lower prevalence of nodules greater than 0.5 mL suggests a relatively small proportion of nodules that are more clinically relevant and require additional diagnostic evaluation or more frequent follow-up.

Aging is a major factor affecting the development of thyroid nodules and multinodularity, as demonstrated by our research and previous studies.^5,7–9^ However, while previous research clearly demonstrates the prevalence of nodules in older people, there are relatively few data on the progression of thyroid nodular disease in younger people. Our results in women of reproductive age show not only a significantly lower proportion and number of nodules compared to the nationally representative population, but also a marked increase in nodules after the age of 25 years, with nodules larger than 5 mm being detected particularly after the age of 40 years. The older age of our pregnant women compared to non-pregnant women of reproductive age could possibly explain the significantly higher prevalence of nodules in the pregnant study group. A recent comparison of pregnant and non-pregnant women, whose ages did not differ, found no significant difference in the number of thyroid nodules.^22^ However, some studies have also shown a significantly higher number of nodules and a larger volume of the dominant nodule in the third trimester compared to the first trimester.^10^,^11^ This suggests that pregnancy-related factors may also influence the development of thyroid nodules.^23^

The possible influence of pregnancy on the development of nodules could be related to the observed higher prevalence of nodules in women, as reported in several studies.^5,8,9,24^ However, some previous reports as well as our results do not show a significantly higher prevalence of thyroid nodules in women, although we demonstrated a higher number of nodules per person in women compared to men.^6^ While we observed a correlation between BMI and thyroid volume in males from the Nutrihealth group and in pregnant women from the EUthyroid study, our results did not confirm an association between BMI and the presence of thyroid nodules in any of the study groups. However, we did find a correlation between BMI and the number of nodules greater than 5 mm. This association has also been observed in some previous studies^8,9,24^ and could be explained by the leptin-induced TSH increase in obese individuals, which leads to TSH-stimulated growth of thyroid follicular cells.^25^

Thyroid volume reflects the iodine supply of a population, with goiter and larger thyroid volume indicating iodine deficiency.^15^ However, the thyroid volumes in our study are consistent with those found in populations with adequate iodine intake.^26^ Compared to areas with iodine deficiency, thyroid volumes in our population are almost half as large in both women and men.^6^ Interestingly, we found no significant difference in thyroid volumes between subgroups of women, despite significant differences in the number and size of nodules. In contrast to previous studies that reported a correlation between thyroid volume and BMI in both females and males,^6^ our study of a nationally representative population confirmed this association only in males from the Nutrihealth study group. However, the significant correlation between thyroid volume and BMI observed in our pregnant women is consistent with the results of previous studies.^10,16^ Our results, as well as others^6,26^, showed a significant negative correlation of thyroid volume and the presence of thyroid nodules with TSH levels in all study populations. This correlation could be attributed to two possible causes. First, without being distinct from other nodules, some nodules may exhibit autonomy, which carries the risk of hyperthyroidism reflected in lower TSH concentrations.^27^ Secondly, in Hashimoto’s thyroiditis, which is very common and often leads to hypothyroidism and higher TSH levels, the thyroid gland may shrink due to tissue destruction.^28^

Although our study results provide valuable insights into the prevalence of thyroid nodules, its limitations must also be emphasized. Firstly, unlike the nationally representative Nutrihealth group, the participants in the EUthyroid study were not nationally representative. The participants were women with no symptoms and no previously known thyroid disease. Therefore, the estismated prevalence in this group reflects the prevalence in a clinically asymptomatic population and not in the general population, as was the case in the Nutrihealth group. Secondly, we did not obtain BMI data for non-pregnant women of reproductive age. Given the significant correlation between BMI and the prevalence of nodules highlighted in some studies, this information would have provided additional insight into the development of nodules in the critical period between the ages of 25 and 40. Finally, we should mention limited number of participants in the Nutrihealth group, where 205 out of 280 people invited to the study responded. With a larger number of participants, we might have gained a better insight into the specific age groups.

In conclusion, it is important to emphasize that the prevalence of thyroid nodules remains high even in areas with adequate iodine intake when high-resolution US is used as diagnostic tool. However, clinically significant nodules are very rare, especially in the younger asymptomatic population, such as our women of reproductive age. The number of nodules increases significantly after the age of 25, while their volume only increases after the age of 40. In asymptomatic individuals with a normal thyroid gland by inspection and palpation, the incidental discovery of small, clinically insignificant nodules during neck imaging, particularly neck US and vascular Doppler, can cause unnecessary anxiety in patients and place an additional burden on the healthcare system.
